# A novel and practical care process framework to inform model of care development

**DOI:** 10.1177/08404704231157215

**Published:** 2023-03-23

**Authors:** Donna Meadows, Joanne Maclaren, Alec Morton, Darcy Ross

**Affiliations:** 1 3527 University of Strathclyde, Glasgow, Scotland, United Kingdom.; 2 8204 Island Health, Victoria, British Columbia, Canada.

## Abstract

Breaking free of pre-existing assumptions to achieve transformative change in care delivery remains challenging. This article presents a care process framework using a rapid task analysis tested with healthcare teams across five communities in British Columbia, Canada, to provide leaders a novel and practical approach to care model development. The study’s goals were to determine if the framework was replicable even though the population care needs differed for each community. The results showed the framework was replicable, informed the care model development, and identified ideal scopes of practice and team composition given the context of care. The framework also captured expert tacit knowledge and decision-making to build capacity given our current workforce challenges. For operational leaders and government agencies, the use of the framework may influence a shift in historical approaches that better aligns health and human resources capacity to population health and service needs.

## Problem

Breaking free of pre-existing assumptions to achieve genuine transformative change in care delivery remains challenging. There is a lack of practical frameworks and explicit designs of studies in the health sciences literature that inspire leaders to think differently. There is also a need to shift from historical approaches to model of care development to one that is more responsive, flexible, and collaborative, where patient and population health needs drive model of care development and align better to a range of scopes of practices and staff mix.

## Background

For more than a decade, healthcare experts across the globe have been warning about the looming health human resource crisis. The COVID-19 pandemic hit a health system already facing multiple challenges: an overworked workforce struggling to keep pace with service expansions in healthcare, magnified by an ageing staff and growing percentage of less experienced clinicians. The shift in workforce composition and demographics has widened an already increasing experience-complexity gap.^1^ Nelson et al. found that the current Canadian system is characterized by insufficiencies in the appropriate and sustainable use of healthcare providers and resources. This is not just a Canadian problem: as Nelson et al. have argued, “The misalignment of Health Human Resources capacities with the need to provide health care services relevant to population demands is a global issue....”^2^ In the report *Defining Health and Health Care Sustainability*, it was stated that the World Health Organization believes somewhere between 20% and 40% of resources spent on health are wasted due to inefficiencies including inappropriate or costly staff mixes.^3^ Having all clinicians work at top of scope or licence just because the regulation allows them to do so without determining the context of the care and the care model in which they function or without addressing a care gap is also problematic. Determining the ideal scopes of practice and roles required to meet the gap(s) for particular care contexts is optimal.

The Rapid Task Analysis (RTA) and the Practice Change Guide (PCG) were two fundamental tools embedded in the development and the refinement of this novel and practical Care Process Framework (CPF). A consistent approach or methodology was needed to capture the tasks (care activities) and the concepts (the education and training required to perform the care activities), involved in addressing the care needs of patients as they journey through a service or program. At the time of this study, a modified Cognitive Task Analysis (CTA) known as an RTA, coined by Goffredson and Mosher in their book *Innovative Performance Support*, was an approach being used within the health authority to build education and learning and performance support resources.^4^ This aligns with the Militello and Hoffman depiction that CTA methods not only help focus on better understanding of the cognitive demands of a task but also on the knowledge and strategies underlying performance.^5^ The RTA was chosen and incorporated into the CPF.

We also learned after the first couple of communities, we tested that implementation of the recommendations was more likely to be successful when a practical and deliberate guide was followed to better articulate to the team the steps involved in implementing practice changes or care model development or re-design. Having a practical framework that maps out the care needs from the patient’s perspective (not the service, professional or provider perspective) created common ground. Seeing how the patient’s care needs shift throughout their care journey helped the leaders (and to a lesser extent their teams) overcome pre-existing assumptions about what care should look like, how it should be delivered, and by which profession. Incorporating a change management approach and engaging teams early and throughout the process was a key learning. Using evidence^1^ and our experience, a practice change guide for leaders to work through when undergoing small to large-scale changes was developed (see Table 1).

**Table 1.** Breakdown of the elements of the Practice Change Guide (PCG) steps.

Steps	Elements
Establish a need	• Identify the drivers • Assess readiness to analyze current/future state • Document and obtain leadership approval
Initiate a plan	• Draft a charter • Obtain leadership approval for the charter • Assess readiness of stakeholders/partners involved • Document and communicate with sponsors
Analyze current state	• Define the patient population (patient care needs) • Review evidence • Review workload and patient care needs data • Assess team’s delivery of collaborative care/communication processes **•** Assess pre-existing assumptions of what care should look like o This will help answer the question, “What may be some of the barriers in redesigning the care model?” • Review technology and/or equipment requirements
Map desired future state	• Articulate expected outcomes o This will help answer the question, “What are the patient/population care needs? Consider future needs • Identify the skills and competencies to address patient care needs o This will help answer the question, “Who can do the work?” • Determine who should do the work • Decide what practice changes or care model changes are required and analyze implications • Draft report with analysis and recommendations/briefing note for decision for practice or care model options • Obtain management review and sponsor sign-off as appropriate • Communicate with stakeholders/partners
Develop implementation (action) plan	• Assign a change leader • Draft project plan • Obtain management review, sponsor sign-off, and permission and resources to implement the plan • Communicate with stakeholders/partners
Implement change	• Assemble the team • Complete the work identified in the plan • Establish/revise standard operating procedures for change(s) • Document progress/status reports as required • Communicate with stakeholders/partners
Consolidate and evaluate change(s)	• Re-enforce the new practice or care model processes/expectations with team following a change management strategy • Evaluate performance metrics • Engage stakeholders/partners as required • Track implementation trends and progress
Sustain and monitor	• Plan and implement a continuous improvement/quality cycle • Identify and respond to emerging issues, risks, and trends • Document and communicate findings with stakeholders/partners/teams • Engage stakeholders/partners and seek assistance as required • Re-initiate process if/when new issues are identified

**Figure 1.**
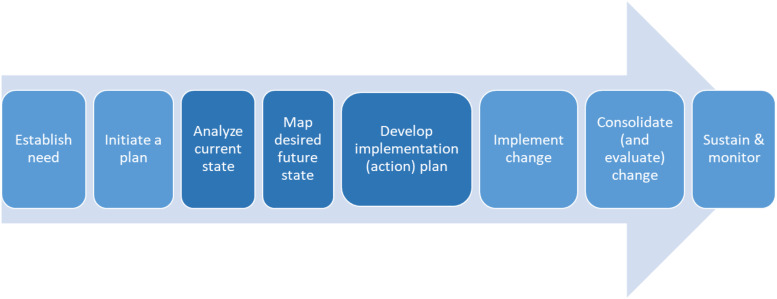
Practice Change Guide (PCG) steps.

The CPF fits within the analyze current state, map desired future state and informing the develop implementation (action) plan (see Figure 1).

## Method

The CPF was tested across five communities as part of a province-wide British Columbia Ministry of Health priority to integrate community-based health services (see Table 2).

**Table 2.** Participating communities.

Communities	Population^2^	Hospital(s)	# beds	Avg. age pop
Mt. Waddington	11,035 (2016)	2 rural, 3+ remote	Rural: 11-bed and 12-bed	41.5
Comox Valley	66,527 (2016)	1 community	146-bed	46.7
Alberni/Tofino	20,712 (2016) 1,932 (2016)	1 community 1 rural	45-bed 10-bed	45.1 38.3
Cowichan Valley	83,739 (2016)	1 community	134-bed	45.7
Saanich Peninsula	114,148 (2017)	1 community	192-bed	48.8

Establish need and initiate a plan were the first steps in the PCG where community leadership reached out, expressed interest, and need, and then were invited to participate. Analyze current state began with our initial review of the local health area profiles. This analysis informed a broader understanding of the population’s demographics and social determinants of health. This included reviewing records from the local hospital Emergency Department (ED) visits to provide insight into the Canadian Triage and Acuity Scale Level (CTAS) range and what individuals in each community were presenting with to gather more insight in the population care needs. Like other ED reviews, up to half of ED presentations are usually manageable outside of the ED.^6^ Map desired future state included structured and semi-structured interviews, observational methods, and think-aloud exercises with Subject Matter Experts (SMEs) which included clinical and non-clinical staff, physicians, and leaders.

**Figure 2.**
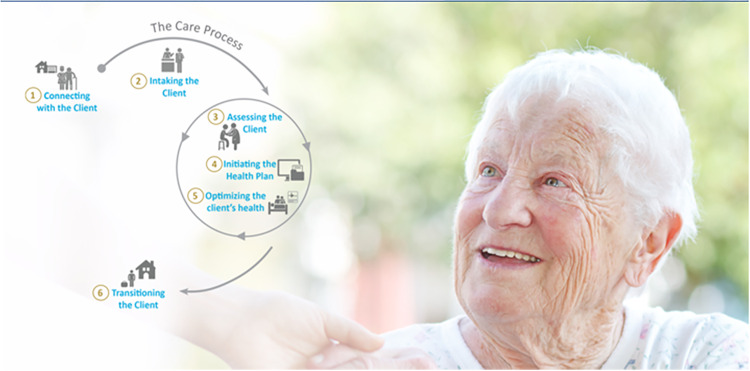
Macro care process for integrated primary and community care.

A care process graphic was created from this engagement with the leaders and SMEs and buckets of like-care activities are grouped together. The “macro care process” represents the care activities or tasks an average or typical patient/client would require while journeying through a service area. For example, patients usually have different care needs depending on where they are at in their health journey mapped across the macro mare process (see Figure 2).

Facilitating dialogue with the SMEs using the RTA then captured the care activities or tasks and steps under each macro care process. Next, each care activity was assigned a risk rating determined by task complexity and risk of failure to perform safely. Tacit decision-making, knowledge, or concepts needed to perform those tasks included relevant legislation, regulation, evidence-based practice standards, training, and decision-support tools. The next steps were to map different professions and roles against those tasks (audience analysis) to determine who could perform those care activities according to their regulatory scopes of practice and/or role or job descriptions, in the case of unregulated care providers. Depending on severity, acuity, intensity, or complexity of care, the care activities are then used to inform ideal scopes of practices and team composition options for that clinical service delivery area given the context of care. The CPF also informs the learning and performance support resources needed to develop and support staff to perform the care activities safely and consistently (see Table 3).

**Table 3.** Sample of the RTA and audience analysis.

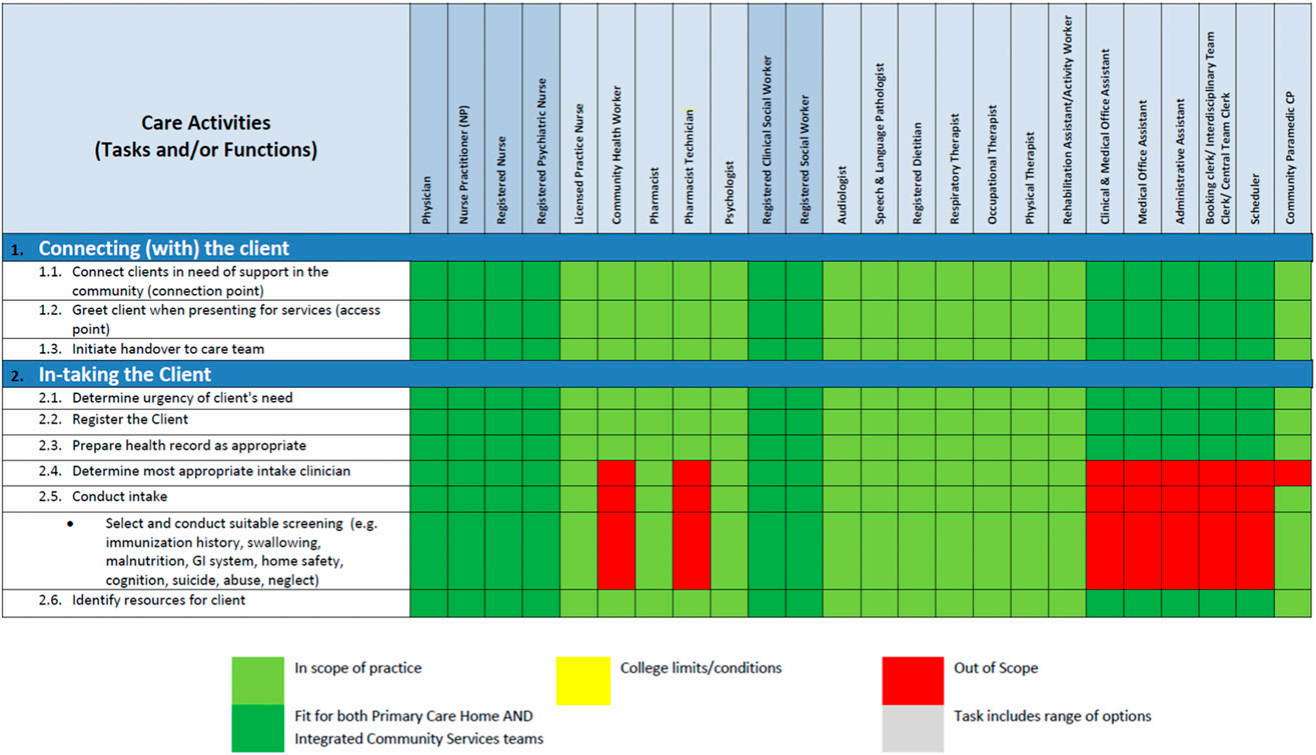

All the data and information gathered from the leaders and the team were then analyzed and a recommendation report was produced. The develop implementation (action) plan was completed in collaboration with the leaders and team after validation and refinement of the information gathered. This enabled the team to identify with the input they provided and contribute to the future state vision. At this point, the CPF team finalized and handed over the final recommendations report to the operational leaders to implement change, consolidate and evaluate change, and sustain and monitor.

## Results

Our experience was that the CPF was replicable as the next team built upon the commonly observed patterns and themes and the learnings were incorporated to strengthen and validate the CPF. Even though each community had differing population care needs, the patient’s overall journey consistently aligned with the same integrated and community care macro care process across the five communities. Additionally, given their knowledge of the patient population, capturing SME clinical decision-making and service delivery expertise, the CPF captured their insights to inform team functioning, optimizing scopes of practice, and determining ideal team composition to address the care, service, and/or practice gaps. SME involvement was critical in identifying the care activities experienced by the patients and informing the possibilities for future care model re-design and better aligning the care to the population care needs. For example, this analysis informed opportunities for members of the team to take on or let go of certain activities and for others on the team to perform care activities at the top of their professional scope or sometimes known as top of licence. Mapping the care activities from the patient perspective and seeing how the patient’s care needs changed depending on where they were at in their health journey, leadership began to shift the assumptions they held about which professions or care team members could address the care needs and when in the care process. This shift in thinking could change how operational leaders and government agencies in the future approach care model development. Two key findings were consistent across five communities:

1) Review of the care activities/tasks and steps in the earlier part of the macro care process (intaking the client, assessing the client, and initiating a health plan with the client) identified opportunities to standardize preventive measures to promote health in high priority areas. Focusing on the right care activities with the right clinicians in the early part of the care process set the stage for the patient to optimize their health and self-management, which can prevent hospitalizations and improve population health.
2) Most care activities the RTA identified within the macro care process were competencies shared amongst multiple members of the interdisciplinary team, and only few were unique to certain professions according to their regulatory scope of practice, role, or job description. This finding presented an opportunity to target scarce education and training resources on shared learning opportunities such as competency development in interprofessional competencies, brief action planning, mental health and substance use, and cultural humility and safety.

In summary, consistent with building on existing strengths, maximizing the potential impact on patient health outcomes, and minimizing the impact of change for staff, all five community recommendation reports included aligning integration of care and resources (such as health human resources, education, and training) to the care process and highlighted key areas to optimize roles, team functioning, and scopes of practice.

## Key success factors

• Having individuals within the local health system who can lead and facilitate framework activities. An objective lens, open perspective, and strong knowledge of the care process framework were invaluable as operational leaders and teams do not always have the objectivity and/or time required to lead this level of quality improvement analysis on their own.
• Like Simken et al., a planning process framework using our PCG with consultation and engagement with leaders and SMEs helped build trust and commitment to carry out the study. Being unfamiliar initially with the new framework, this trust building was a critical element to not only capturing the true current state and the barriers to service delivery encountered but also in providing recommendations that resonated with the team.^7^
• The RTA family of methods provides strength to our design as it has a longstanding history and evidence of growing use ^8-16^ and untapped potential in the health sciences research.^17^
• Aligning the RTA with the care process to capture expert and tacit knowledge and decision-making provided the novel design element Graham et al. state is missing from the health and implementation sciences literature.^18^

## Discussion

Now more than ever, the need to have a practical and evidence-informed approach to designing care models is required given the shifting healthcare environment and sustained health human resource challenges. With the complexity of healthcare, it is imperative to have a practical framework leaders can use to develop new care models that are aligned and driven by patient and population health needs.

There is also a need to shift approaches when determining care models given the current number of employees and high demand for experienced staff. As Stevenson et al. state: “We must go beyond traditional approaches and challenge outdated beliefs that we can recruit our way out of this situation.”^19^ Yet many leaders conceptually approach new care model development with a focus on status quo or a strong pre-existing mindset about what service delivery “should” look like. This is often informed by individual experiences even when the existing models are not consistently effective. These model changes generally “add more” professions to the team without fully optimizing the professions they already have. Innovations in policy, planning, and funding must align and support changes in care model development and health service planning must be aligned with health human resource planning.^2-3, 20^ In many ways, the COVID-19 pandemic and health human resource challenges we are currently experiencing have inspired some much-needed innovative thinking in care model development.

## Lessons learned

Our experiences consistently revealed four key lessons for scholars and leaders in this area:

• The need for strong executive leadership and sponsorship to drive engagement with the care teams.
• The conversations and recommendations related to redesigning the team and/or performance expectations challenged the existing culture of independent practice; optimizing existing staff to top of scope of practice or licence requires change leadership resources as does adding a new role, building team competency, or implementing a change in team processes.
• Building a care process and mapping the care activities using the RTA illuminated a valuable and deeper understanding of the care being provided, and only after the framework unfolded, were leaders/teams able to envision and inform a future model of care.
• For operational leaders and government agencies, using the care process framework could represent a departure from engrained historical thinking about “add more” vs. “add right” health human resources, education, and training allocation strategies.

## Conclusion and ongoing work

There is a need to meet our population’s healthcare needs effectively and optimally given the health and human resources available. This paper presents a novel care process framework that can inform models of care development and help leaders break free of pre-existing assumptions to achieve transformative change in care delivery. Our framework is a practical one that is replicable across service or program areas despite differing population care needs. The novel care process framework (a) informs new thinking around care model development, scope of practice, and team optimization in the context of that environment or care setting and (b) captures expert tacit knowledge to support novice decision-making, and so would intuitively expedite training and onboarding and further improve quality and care outcomes. Further application of our framework in other service areas is resulting in comparable results.

## Notes

1. PROSCI©, John Kotter’s 8 step Change Model Methodology and The PEPPA Framework (McMaster) were used to inform our Practice Change Guide framework.

2. Statistics Canada (2016) Census Profile, 2016 Census—Vancouver Island and Coast [Economic region], British Columbia and British Columbia [Province] (statcan.gc.ca).
